# COVID‐19‐vaccinated plasma treatment for COVID‐19 patients?

**DOI:** 10.1111/irv.12852

**Published:** 2021-03-14

**Authors:** Gabriela Gama Freire Alberca, Ricardo Wesley Alberca

**Affiliations:** ^1^ Laboratorio de Dermatologia e Imunodeficiencias (LIM‐56) Faculdade de Medicina FMUSP Departamento de Dermatologia Universidade de São Paulo São Paulo Brazil

## PEER REVIEW

The peer review history for this article is available at https://publons.com/publon/10.1111/irv.12852.

*Dear Editor:*

Recently, we published a letter entitled “What is the long‐term clinical significance of anti‐SARS‐CoV‐2‐specific IgG?” about the rapid decay total levels and neutralizing anti‐SARS‐CoV‐2 antibodies in asymptomatic and mild patients.^1^ Nevertheless, with the increase in vaccination worldwide, we would like to add a few comments to our previous letter.

Post‐infection patients develop increased antibodies against two major SARS‐CoV‐2 viral proteins, spike, and nucleocapsid.^2^ Since the beginning of the pandemic, passive immunization with plasma derived from patients recovered from COVID‐19 has been used as a virus‐specific therapeutic intervention. A recent randomized trial with 288 patients hospitalized with severe COVID‐19 pneumonia verified no significant differences in the clinical status or mortality in the treatment with placebo or convalescent plasma.^3^ A possible explanation is that the levels of neutralizing antibodies and the duration that anti‐SARS‐CoV‐2 antibodies levels remain high for a very short period after natural infection and antibody production is dependent on the severity of the infection.^4^ Therefore, these factors may affect the efficiency of the treatment with convalescent plasma. Alternatively, the usage of anti‐SARS‐CoV‐2 monoclonal antibodies to treat COVID‐19 patients presented promising results, but it may be limited by the costs and production capacity.^5^ With the beginning of the vaccination process worldwide, we would like to propose an alternative to the convalescent plasma: the vaccinated plasma.

With the vaccination process, the numbers of cases and deaths by COVID‐19 are expected to drop, once “herd immunity” is established. Nevertheless, due to the limitations of the overall production and distribution of the vaccines, this process could take years. Therefore, due to the current health, economic and social disruption caused by the pandemic^6^, ^7^ investigating treatments for COVID‐19 patients is a necessary process.^8^, ^9^ Vaccination produces a safe and effective immune response to COVID‐19, with high titers of neutralizing anti‐SARS‐CoV‐2 IgG antibodies.^10^

Therefore, the use of vaccinated plasma could present an important, safe, and more effective intervention in comparison with convalescent plasma. Indeed, the effectiveness of this procedure still needs to be investigated, as well as proper dosage and timing of the plasma transfer in relation to the infection day. Nevertheless, we believe that this could represent a promising strategy in the current pandemic situation, which should be discussed by the audience of this prestigious journal.

## CONFLICT OF INTEREST

All authors have no conflicts of interest to declare.

## AUTHOR CONTRIBUTIONS

**Gabriela Gama Freire Alberca:** Visualization (equal); Writing – original draft (equal); Writing – review and editing (equal). **Ricardo Wesley Alberca:** Conceptualization (lead); Supervision (equal); Visualization (equal); Writing – original draft (equal); Writing – review and editing (equal).

## References

[1] Alberca GGF , Alberca RW . What is the long‐term clinical significance of anti‐SARS‐CoV‐2‐specific IgG? Influenza Other Respi Viruses. 2020;1‐2. 10.1111/irv.12825 PMC8051736 33131215

[2] Xiao C , Ling S , Qiu M , et al. Human post‐infection serological response to the spike and nucleocapsid proteins of SARS‐CoV‐2. Influenza Other Respi Viruses. 2021;15(1):7‐12. 10.1111/irv.12798 PMC7461388 32844604

[3] Simonovich VA , Burgos Pratx LD , Scibona P , et al. A randomized trial of convalescent plasma in Covid‐19 severe pneumonia. N Engl J Med. 2020;384(7):619‐629. 33232588 10.1056/NEJMoa2031304 PMC7722692

[4] Bošnjak B , Stein SC , Willenzon S , et al. Low serum neutralizing anti‐SARS‐CoV‐2 S antibody levels in mildly affected COVID‐19 convalescent patients revealed by two different detection methods. Cell Mol Immunol. 2020:1‐9. 10.1038/s41423-020-00573-9 PMC7604543 33139905

[5] Baum A , Fulton BO , Wloga E , et al. Antibody cocktail to SARS‐CoV‐2 spike protein prevents rapid mutational escape seen with individual antibodies. Science. 2020;369(6506):1014‐1018. 32540904 10.1126/science.abd0831 PMC7299283

[6] Ismail SA , Huntley C , Post N , et al. Horses for courses? Assessing the potential value of a surrogate, point‐of‐care test for SARS‐CoV‐2 epidemic control. Influenza Other Respi Viruses. 2021;15(1):3‐6. 10.1111/irv.12796 PMC7436809 32767548

[7] Lim JM , Tun ZM , Kumar V , et al. Population anxiety and positive behaviour change during the COVID‐19 epidemic: Cross‐sectional surveys in Singapore, China and Italy. Influenza Other Respi Viruses. 2021;15(1):45‐55. 10.1111/irv.12785 PMC7767950 32889784

[8] Alberca RW , de Souza Andrade MM , Castelo Branco ACC , et al. Frequencies of CD33+ CD11b+ HLA‐DR‐ CD14‐ CD66b+ and CD33+ CD11b+ HLA‐DR‐ CD14+ CD66b‐ cells in peripheral blood as severity immune biomarkers in COVID‐19. Front Med. 2020;7:654. 10.3389/fmed.2020.580677 PMC7592395 33178720

[9] Alberca RW , Teixeira FME , Beserra DR , et al. Perspective: the potential effects of naringenin in COVID‐19. Front Immunol. 2020;11:570919. 33101291 10.3389/fimmu.2020.570919 PMC7546806

[10] Zhang Y , Zeng G , Pan H , et al. Safety, tolerability, and immunogenicity of an inactivated SARS‐CoV‐2 vaccine in healthy adults aged 18–59 years: a randomised, double‐blind, placebo‐controlled, phase 1/2 clinical trial. Lancet Infect Dis. 2020;21(2):181‐192. 33217362 10.1016/S1473-3099(20)30843-4 PMC7832443

